# An entropic explanation of insistence on sameness in autism

**DOI:** 10.3389/fncom.2025.1714428

**Published:** 2026-01-27

**Authors:** Przemysław Śliwiński

**Affiliations:** Faculty of Information and Communication Technology, Wrocław University of Science and Technology, Wrocław, Poland

**Keywords:** autism definition, entropy distance, insistence on sameness, robotized live-in caregiver, therapeutic algorithms, therapy design guidelines, uncertainty minimization

## Abstract

**Purpose:**

An information theory-based framework is proposed in attempt to explain *insistence on sameness in autism* as an instance of a general behavior pattern in which an individual tries to reduce surprise and uncertainty. It offers a new definition of autism as *an impairment in which cognitive functions are restricted to discrimination, memorization and prediction of tangible properties of the environment*.

**Methods:**

An analogy between *insistence on sameness* and *constrained minimization* of the *entropy metric* is observed and examined for a set of assumptions that describe cognitive limitations of a person with autism. The metric is given by the formula *D*_*H*_(*R, M*) = *H*(*R*|*M*)+*H*(*M*|*R*), where *R* represents sequences of random stimuli, *M* is a memory that stores and retrieves them, and where *H*(·|·) denotes their *conditional entropies* interpreted as *surprise* and *uncertainty*, respectively.

**Results:**

It is first inferred that to minimize the metric an individual can learn about *R* (and store that knowledge in *M*) or can restrict *R* to the already known *M*. Then, it is concluded that *insistence on sameness* is a manifestation of the latter. Moreover, it is shown that the proposed framework: (1) Helps to quantify the concepts of *surprise, uncertainty, sensory overload and deprivation, anxiety, comfort zone, disappointment, disorientation, pedantry, rigidness, observance* or *aberrant precision*. (2) Leads to a list of guidelines for learning therapies and daily care routines, and allows them to be defined as optimization algorithms and implemented as programs for *robotic live-in caregivers*. (3) Can be validated with the help of a *Turing test*-like approach that requires no experiments involving individuals with autism.

**Conclusion:**

The framework—if positively validated—will provide advantages of both theoretical and practical importance: it explains the insistent on sameness as a consequence of cognitive restrictions and offers formal foundations and design guidelines for therapies aimed at improving *self-reliance* of individuals with autism in *basic activities of daily living*.

## 1 Introduction

Etiology and pathogenesis of autism remain unclear as does its definition (Wiggins et al., 2009; American Psychiatric Association, DSM-5 Task Force, 2013; Davis and Plaisted-Grant, 2015; Sauer et al., 2021; Volkmar, 2021; Jourdon et al., 2023; Shiraishi et al., 2024; Assary et al., 2024; Mahé et al., 2025). There is no a consensus about the behavior traits that are associated with individuals with autism either (Mottron and Bzdok, 2020; Chapman and Veit, 2021; Perochon et al., 2023). While behavior, in general, appears to be driven by a variety of templates and patterns (see e.g., Lai, 2023 and *cf*. Bailey et al., 2024), we focus on *insistence on sameness*, a phenomenon referred also to as *stereotyped, repetitive*, and the *autistic-like behavior* or the *autism-like trait* (*cf. e.g.*, Uljarević et al., 2017; Volkmar, 2021; Lyu et al., 2024; Kamp-Becker, 2024) observed in non-verbal low-functioning individuals (the term *low-functioning* is used to denote *severe* (*profound*) instances of the *Level 3 of Autism Spectrum Disorder, cf*. American Psychiatric Association, DSM-5 Task Force, 2013; Betty et al., 2015; Wang et al., 2022; Singer et al., 2023).

We propose a new insight into that phenomenon and examine its resemblance to a problem of *constrained minimization of uncertainty*, where the following *entropy metric* (see *e.g.*, MacKay, 2003):


$$\begin{array}{l} D_{H}\left(R,M\right)=H\left(R|M\right)+H\left(M|R\right), \end{array}$$ (1)

is used as a *measure of uncertainty*. Random variables, *R* and *M*, represent the real-world environment and the memory of an individual, respectively. The terms *H*(*R*|*M*) and *H*(*M*|*R*) are *conditional entropies* that quantify the levels of *surprise* and *uncertainty*.

The motivation for such an approach is to devise a mathematically rigorous framework that offers an *explanation* of the insistence on sameness which is more precise and less arbitrary than the now dominant language-based descriptions (*cf. e.g.*, American Psychiatric Association, DSM-5 Task Force, 2013; Mottron and Bzdok, 2020; Chapman and Veit, 2021; Volkmar, 2021; Wang et al., 2022), enables formulation of learning therapies as solutions to a constrained optimization problem and facilitates their implementations as programs for *e.g.*, robot-based daily life assistants.

The framework is inspired by a variety of results and observations from information theory, neuroscience and psychiatry (see Table 1) and its contribution is twofold, *theoretical* (a relatively simple and verifiable explanation of *insistence on sameness* as a consequence of cognitive restrictions of a person with autism) and *practical* (a set of design guidelines for therapies focused on *low-functioning non-verbal* individuals), and may lead to a more precise definition of the disorder and better personalized therapies.

**Table 1:** Works that led to the development of the framework.

**References**	**Related scope**
MacKay, 2003	An accessible information theory textbook, where basic definitions of the *entropy metric, conditional and relative entropies* are introduced and derived.
Friston, 2010	A review paper that discusses a hypothesis that the *free-energy principle* can be a basis for a universal brain theory.
American Psychiatric Association, DSM-5 Task Force, 2013	A diagnostic manual that presents diagnostic criteria, verbal descriptions of behavior traits associated with autism (incl. *insistence on sameness*); definitions of the *autism spectrum disorder severity levels 1-3*.
Lawson et al., 2014	Introduction, analysis and early experimental verification of a link between *free-energy principle* and *aberrant precision* phenomenon in autism.
Galitsky, 2016	An attempt to create a computational model of autism perception based on mostly verbal description of the functional *theory of mind*.
Davis and Plaisted-Grant, 2015	Discussion of a possibly multifold (positive and negative) impact of noise on autism traits and the associated cognitive abilities.
Palmer et al., 2017	Reviews Bayesian approaches to autism. Introduces a model based on assumption that underlies a significant impact of the sensory information on building and updating the internal representation of environment.
Uljarević et al., 2017	A study that links *insistence on sameness* with *self-regulation* in autism. It also verifies experimentally its relationship with *effortful control* and *anxiety* phenomena.
Mottron and Bzdok, 2020	A proposal of several remedies to the problem of multifold increase in autism diagnoses, incl. narrowing its definition and combining the experience of clinicians with qualitative features.
Volkmar, 2021	A comprehensive, extensive and detailed compendium describing autism spectrum disorder-related topics; in particular, *low-functioning individuals* and *insistence on sameness*.
Wang et al., 2022	A survey presenting *state-of-the-art* results in the areas of autism diagnosis and intervention. Prioritizes *functional-based* therapeutic tools.
Ramstead et al., 2023	A review paper which discusses in a formalized fashion the universality of *free-energy principle* and explains its relationship to other principles and laws of physics and information theory.

Clearly, there are some limitations of the framework: it is a *functional* model, *i.e.*, it does not define the underlying biological implementation. Is also requires new and carefully designed personalized validation experiments to be developed. In turn, limiting the scope of the framework to low-functioning non-verbal individuals with autism is a deliberate attempt to make it both formal and accurate.

In the following sections we present the framework, examine its basic properties and discuss its relations with insistence on sameness. Then, we devise the guidelines for learning therapies aimed at increasing autonomy of persons with autism, formulate a therapy as an optimization problem and discuss two framework validation approaches. Finally, we conjecture that *insistence on sameness* is a special case of a general minimizing uncertainty pattern (*cf*. Friston, 2010) and conclude the work with the proposal of a new definition of autism.

## 2 Methods

**Figure 1:**
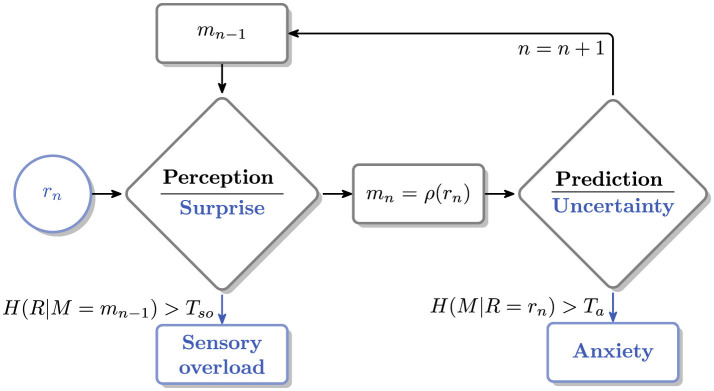
Stimuli processing loop.

The framework consists of three components: the entropy metric (1), a stimuli processing loop in Figure 1, and a set of Assumptions 1 –4 that represent the cognitive constraints of a person with autism.

### 2.1 Entropy metric

The metric (1) measures an *entropy distance* between two discrete random variables *R* and *M*. To illustrate its basic properties, we will consider two opposite scenarios:

1. *R* fully determines *M* (and *vice versa*). In this case knowledge of one variable removes the entire uncertainty about the other and thus both conditional entropy terms are zero (and so is the metric),
  $$D_{H}\left(R,M\right)=\underset{=0}{\underset{︸}{H\left(R|M\right)}}+\underset{=0}{\underset{︸}{H\left(M|R\right)}}=0.$$
2. Both *R* and *M* are independent of each other. Knowing either of them carries no information about the other (so they maintain their original uncertainties):
  $$D_{H}\left(R,M\right)=\underset{=H\left(R\right)}{\underset{︸}{H\left(R|M\right)}}+\underset{=H\left(M\right)}{\underset{︸}{H\left(M|R\right)}}=H\left(R\right)+H\left(M\right).$$

Hence, in order to reduce the entropic distance between *R* and *M* one can learn about *R* (and store that knowledge in *M*), or constrain *R* to the already known *M*.

### 2.2 Stimuli processing loop

The loop (see Figure 1) serves as a model of stimuli processing. Its two phases, *perception*, and *prediction* are associated with the components of the metric, *H*(*R*|*M* = *m*_*n*−1_) and *H*(*M*|*R* = *r*_*n*_), that quantify how *surprise* and *uncertainty* are affected by the incoming stimulus *r*_*n*_. If levels of surprise or uncertainty exceed the respective thresholds, *T*_*so*_ or *T*_*a*_, a *sensory overload* or *anxiety* state can occur.

### 2.3 Assumptions

To specify the cognitive constraints of a non-verbal low-functioning individual with autism, we assume that:

1. The *stimuli*, *r*_*n*_, *n* = 0, 1, …, represent a perceived environment and form sequences. The mixture of all sequences is denoted by *R*.
2. A memory, *M*, is capable of storing sequences and retrieving their elements, *m*_*n*_, *n* = 0, 1, ….
3. Each memory item, *m*_*n*_ = ρ(*r*_*n*_), is a result of a classification of a single stimulus, *r*_*n*_, by a *nearest neighbor algorithm*.
4. Both *sensory overload* and *anxiety* thresholds, *T*_*so*_, *T*_*a*_>0, are random variables.

### 2.4 Comments on assumptions

**Figure 2:**
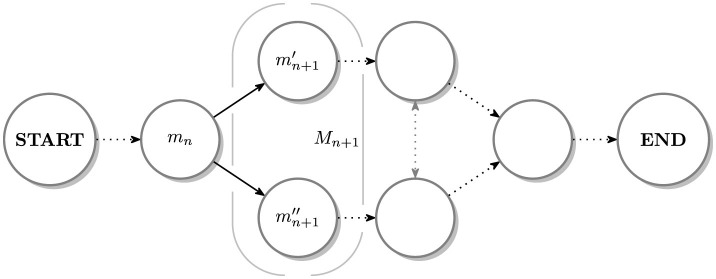
An instance of a graph composed of memorized stimuli sequences, *m*_*n*_ = ρ(*r*_*n*_), *n* = 0, 1, … with a set $M_{n+1}=\left\{m_{n+1}^{\prime },m_{n+1}^{\prime \prime }\right\}$ consisting of a pair of alternative events.

Each stimulus, *r*_*n*_, can be interpreted as a “*snapshot*” of the environment and *m*_*n*_ = ρ(*r*_*n*_) as its counterpart perceived by the senses and located in memory *M* (*cf. e.g.*, Nobre, 2022). The sequences in the memory are represented by chains/directed graphs (see Figure 2).

Remark 2.1. All stimuli are treated in a unified way, as raw (random) signals, and their sources (*e.g.*, external/exogenous or internal/endogenous) are not distinguished. Moreover, their semantics (*i.e.*, the non-tangible, abstract, cultural meanings and associations) are not available. Thus, only their perceptible properties are taken into account and treated as equally important; *i.e.*, all their abstract properties and relations (see *e.g.*, Bäck, 2014), incl. *importance, hierarchy, being a part/whole* are ignored.

Example 2.1. An abstract notion of *nothing* cannot be represented by any perceptible stimulus. This observation can also be derived from the namesake property of a *nearest neighbor algorithm* (*cf*. Assumption 3), where the classification routine always selects the closest remembered (known) item, regardless of how "far" it is in terms of a distance function.

Remark 2.2. Assumptions 2–3 define a cognitive model with the inference abilities limited to replication of the memorized stimuli and their sequences. They also imply a non-vanishing (persistent) character of the memory.

Remark 2.3. Defining the thresholds, *T*_*so*_, *T*_*a*_, as random variables allows them to be unknown, varying in time, specific for an individual and driven by the factors not included in the model (*cf. e.g*. Kim and Kim, 2023; Lai, 2023).

### 2.5 Analysis

The goal of this section is to inspect how the metric changes during *perception* and *prediction* phases; *cf*. the processing loop in Figure 1. The amount of *surprise* in the former, given the memorized stimulus, *m*_*n*−1_, is


$$\begin{array}{l} D_{H}\left(R,M|M=m_{n-1}\right)=H\left(R|M=m_{n-1}\right)+\underset{=0}{\underset{︸}{H\left(M=m_{n-1}|R\right)}}, \end{array}$$ (2)

while the level of *uncertainty* in the latter, given the incoming stimulus, *r*_*n*_, is


$$\begin{array}{l} D_{H}\left(R,M|M=r_{n}\right)=\underset{=0}{\underset{︸}{H\left(R=r_{n}|M\right)}}+H\left(M|R=r_{n}\right), \end{array}$$ (3)

so that during *perception* we need to consider only the first term, *H*(*R*|*M* = *m*_*n*−1_), and only the other, *H*(*M*|*R* = *r*_*n*_), during *prediction*. We examine them in the following three cases; *cf*. Section 2.1:

1. The first, *deterministic*, takes place when in *perception* phase knowledge of *m*_*n*−1_ determines *r*_*n*_ so that there is no *surprise*, and when there is no *uncertainty* during *prediction*, because knowledge of *r*_*n*_ determines *m*_*n*+1_. Then, both terms are zero:
  $$\begin{array}{l} H\left(R=r_{n}|M=m_{n-1}\right)=0\text{ and }H\left(M=m_{n+1}|R=r_{n}\right)=0. \end{array}$$ (4)
2. The second case occurs when, in the *perception* phase, *m*_*n*−1_ determines a set *R*_*n*_ of possible stimuli or, when in *prediction*, the set of memorized possibilities *M*_*n*+1_ occurs given the stimulus *r*_*n*_ (*cf*. Figure 2). The respective metric terms are:
  $$\begin{array}{l} H\left(R=R_{n}|M=m_{n-1}\right)=H\left(R_{n}\right)\text{ and}\\ H\left(M=M_{n+1}|R=r_{n}\right)=H\left(M_{n+1}\right). \end{array}$$ (5)
3. The last case encompasses, amongst others, the learning-like situations when *r*_*n*_ is a new (unknown) stimulus, so that the current knowledge of *m*_*n*−1_ carries no information about it during *perception* and the stimulus itself does not reduce the level of *prediction uncertainty*:
  $$\begin{array}{l} H\left(R|M=m_{n-1}\right)=H\left(R\right)\text{ and }H\left(M|R=r_{n}\right)=H\left(M\right). \end{array}$$ (6)

It should be noted here that because the sets of known options, *R*_*n*_ and *M*_*n*+1_, are subsets of *R* and *M*, the amounts of *surprise* or *uncertainty* in 2 seem to always be smaller than in 3 (when unknown stimuli occur). Nevertheless, for some realizations of *m*_*n*−1_ and *r*_*n*_ the following inequalities may hold (see *e.g.*, MacKay, 2003, Ch. 8):


$$\begin{array}{l} H\left(R_{n}|M=m_{n-1}\right)\geq H\left(R\right)\text{ and }H\left(M_{n+1}|R=r_{n}\right)\geq H\left(M\right), \end{array}$$ (7)

and hence the *surprise* and *uncertainty* levels in (5) can be larger than in (6).

Example 2.2. Such a situation can take place when, in *prediction* phase, the set *M*_*n*+1_ consists of multiple options, $\left\{m_{n+1}^{\prime },m_{n+1}^{\prime \prime },\ldots ,m_{n+1}^{\left(\nu \right)}\right\}$, some ν, that are equally probable; (*cf*. Figure 2). The amount of *uncertainty* is then increased (predictions are the least efficient) and can easier lead to *disorientation* and *anxiety* (especially when ν is large).

So far, we have conditioned the metric terms on the last realizations of the stimulus *r*_*n*_ (or on the latest memory item *m*_*n*−1_), as if the amounts of *surprise* and *uncertainty* are independent of the past [like in Markov chains (see *e.g.*, Ekroot and Cover, 1993)]. However, to better comply with Assumptions 2 –3 and Remarks 2.1–2.2, one should take into account the previous events as well.

Example 2.3. If, for instance, in the *perception* phase, we condition the *surprise* on the set *M*_*n*−1_ = {*m*_*n*−1_, *M*_*n*−2_, …} of all previous items in a sequence, then the term *H*(*R*|*M* = *M*_*n*−1_) can quantify a *disappointment* that the sequence does not continue in a way it has been memorized.

#### 2.5.1 Classification

The influence of the *classification* phase (*i.e.*, the assignment *m*_*n*_ = ρ(*r*_*n*_) in Figure 1) has so far been ignored. This is because it resembles the impact of *prediction*. First, observe that the metric reduces to the same term; *cf*. (3):


$$D_{H}\left(R=r_{n},M\right)=\underset{=0}{\underset{︸}{H\left(R=r_{n}|M\right)}}+H\left(M|R=r_{n}\right).$$

Next, note that if the stimulus, *r*_*n*_, is already known, then it fully determines the corresponding *m*_*n*_ (*cf*. Assumption 2 and Remarks 2.1–2.2). In turn, if *r*_*n*_ is new, it carries no information about the memory. Hence, *cf*. (4) and (6):


$$H\left(M|R=r_{n}\right)=\{\begin{array}{l} 0\\ H\left(M\right) \end{array}\text{ if }r_{n}\text{ is }\begin{array}{l} \text{known,}\\ \text{new.} \end{array}$$

Remark 2.4. Although the presence of randomness, even in a fixed and arranged environment, seems to be inevitable (*cf. e.g.*, Davis and Plaisted-Grant, 2015; Walker et al., 2023), the modified environments and/or the sequences can still be perceived as known as long as those random changes do not affect the results of classification; *cf*. Remark 2.1.

## 3 Results

### 3.1 Analogies with insistence on sameness

In case of non-verbal low-functioning individuals with autism, both *sensory overload* and *anxiety* can lead to violent, (self-)aggressive behaviors [sometimes labeled as *meltdowns* or *tantrums* (Volkmar, 2021)]. Insistence on sameness can be seen as a set of actions aimed at decreasing the risk of these events, either by observing (learning) the environment (*i.e.*, making *M* better approximate *R*), or by constraining it to the already known one (that is, making *R* better resemble *M*). For example (*cf*. Uljarević et al., 2017):

1. *Pedantry*, together with a *routinized* and *repetitive* behavior [inc. strict following only known sequences of activities and keeping the known environments unchanged; *cf*. an *aberrant precision* in (Lawson et al., 2014)], are the means to reduce the levels of surprise or uncertainty; see Ex. 2.2, 2.3 and Remark 2.4.
2. *Ritualistic* and *stereotyped* behaviors stem from the lack of the ability to ignore items; see Remarks 2.1-2.2 and *cf*. Guideline 3.
3. *Restrictive* and *rigid* behavior includes actions like forcing deterministic scenarios in order to further control *surprise* and *uncertainty* [and to effectively zero their levels as in (4)], to avoid *disappointments* and to select a preferred (the most frequent) option in multiple option cases; *cf*. (5), (7) and Ex. 2.3.
4. *Vigilance* and *observance* reduce the number of options, that is, the uncertainty level caused by them in (5); see Ex. 2.2.
5. Aversion to learning is a way to avoid potentially high surprise/uncertainty levels caused by new options or new stimuli; *cf*. (5)-(7).

### 3.2 Therapeutic guidelines

In practical terms, the goal of the learning therapy is twofold:

1. Increasing autonomy of an individual (by learning new vital activities in a supervised way),
2. Reduction of aversion to exploration (by controlling uncertainty of the new environments designed for semi-supervised and unsupervised activities).

Assumptions 1-4, Remarks 2.1–2.3, and the subsequent analysis in Section 2.5, imply that the therapy is a long-term incessant process with several limitations on how it can be implemented (in particular, they exclude rule-based learning; *cf*. Remarks 2.1-2.2 and Betty et al., 2015; Galitsky, 2016):

1. Only tangible objects and their realistically simulated counterparts should be used in learning environments and in sequences of activities.
2. Artifacts such as pictures (especially, in simplified forms of drawings or labels), but also spoken words and sentences, should *a priori* be assumed as unrelated with the tangible items they represent or refer to. Hence, if necessary, these relations need to be learned separately. Abstract notions, like being a child or a parent, a teacher or a caretaker, and relations like love, trust or friendship, are not available either.
3. The meaning of tangible items depends on the context (created by other items and/or their past sequences; *cf*. Remark 2.2). Such a correlative-like reasoning leads to ”*cum/post hoc ergo propter hoc*” fallacies; see Example 3.2.
4. Remembering the exact sequences (of events and activities; *cf*. Remarks 2.1 and 2.2) implies they are treated as a whole and that ”*shortcuts*” in sequences or other ”*by-the-way*” activities will likely be perceived as new (and will increase levels of surprise and/or uncertainty); *cf*. Ex. 2.3 and (Lawson et al., 2014; Noel et al., 2025).
5. Multiple choice situations (branches in sequences; see Figure 2) introduce uncertainty even if the environment remains unchanged; *cf*. Cases 2–3, and Exs. 2.2–2.3 and 3.1.

Example 3.1. In order to reduce the risk of anxiety induced by predictions in multiple option/choices situations, several countermeasures could be applied: making all sequences distinct (with the help of *amulet- or talisman-like* artifacts that will allow to distinguish the otherwise same activities), or locating the branches at the sequence beginnings (to reduce these uncertainties as early as possible), or limiting the numbers of possible options in them.

Example 3.2. An instance of an immediate reward will likely turn into a part of a routine while its delayed version will become a part of (possibly unrelated) sequence it will occur in. This is because the delayed gratification concept relies on an abstract concept of an award and its causal relation with the past events.

### 3.3 Self-stimulation activities and comfort zone

The guidelines 1–5 are aimed at reducing risk of tantrums during learning/therapy. However, in case of non-verbal individuals, it is difficult to assess whether the levels of surprise and/or uncertainty are sufficiently low (w.r.t. their thresholds *T*_*so*_and*T*_*a*_) to safely start a new sequence of activities. The problem is of special importance at the beginning of the therapy when the environments and activities the individuals are familiar with (inc. their routines stored in *M*) are not known. Nevertheless, if the stimuli are treated in a unified way, so that their exogenous or endogenous nature is not distinguished (*cf*. Assumption 1 and Remark 2.1), we can conjecture that (*cf*. Davis and Plaisted-Grant, 2015):

Conjecture 3.1. *Self-stimulation* activities [inc. ”*flapping, waving, finger wiggling, mouth opening, orofacial movements, head nodding”* (see Volkmar, 2021)], are a sign of low levels of perceived surprise and uncertainty.

The rationale behind this claim is following: without external stimuli (in a state of *sensory deprivation*), the internal ones become dominant. If sufficiently random, they can carry an amount of entropy large enough to exceed either sensory overload or anxiety threshold. *Self-stimulations* can therefore (in case of low-functioning individuals, in particular) be a way to generate known (zero- or low-entropy) stimuli that keep the surprise/uncertainty levels below these thresholds (*i.e.*, inside a ”comfort zone”).

Remark 3.1. One of the Reviewers raised a question about the role of *white noise*. That is an interesting and open problem as one can consider two potentially opposite, *negative* and *positive*, effects:

1. Presence of a white noise in stimuli increases their conditional entropy and the risk of *sensory overload*. It can affect the results of the classification and increase the risk of *anxiety* as well; *cf*. Remark 2.4.
2. In turn, as pointed out by (Davis and Plaisted-Grant 2015), noise can help avoid getting stuck in local minima during optimization and, for instance, enable generalization (abstraction) abilities. In our framework, such effect can be associated with jumping between the known sequences or finding the shortcuts within them.

White noise, if present in exogenous visual or aural stimuli, could be detected with the help of basic audio-video codecs [the more noisy environment, the less effective entropy coders; (see MacKay, 2003, Ch. 1)].

### 3.4 Learning therapy as optimization problem

In the framework's terms, learning a low-functioning person with autism is equivalent to presenting new stimuli and their sequences. Therefore, a learning therapy with the goals 1–2, can be formulated as the following optimization problem: Problem 2.1. Given an initial state of a memory *M*, find *R* such that


$$\begin{array}{l} \underset{R}{\arg\max}\;I\left(R;M\right), \end{array}$$ (8)

subject to


$$H\left(R|M=m_{n-1}\right)<T_{so}\text{ and }H\left(M|R=r_{n}\right)<T_{a},$$

given Assumptions 1–4 and the processing loop in Figure 1.

The *objective function* (8) is the *mutual information* of *R* and *M*, and is related to the entropy metric in (1) via identity *D*_*H*_(*R, M*) = *H*(*R, M*)−*I*(*R*; *M*), where *H*(*R, M*) is the joint entropy of *R* and *M* (see MacKay, 2003, Ch. 8; and Friston, 2010; Ramstead et al., 2023). The practical value of such a formulation is that it allows learning therapies and daily care routines to be defined as optimization algorithms and implemented as programs for *robotic live-in caregivers*.

## 4 Discussion

### 4.1 Validation

One can consider the following two (complementary) approaches to validate/reject the framework's assumptions and the resulting analogies:

1. A traditional (direct) variant, where the realistic environments and interactively generated sequences of new activities are presented through a “digital window” (*e.g.*, a wall-like display with a haptic interface). The intensity (and the pace the sequences are presented with) depends, in accordance to recommendations 1 –5, on the levels of surprise and anxiety assessed from *e.g.*, the observed behavior or from a frequency of interactions between the individual and the generated world. The long-term therapeutic effects, like increased autonomy in these environments (with possible help of appropriately programmed robotic live-in caregivers), is evaluated in a traditional way (*e.g.*, in a form of progress questionnaires) by parents and therapists.

This is a standard approach that requires an access to individuals with autism living in their homes (or other familiar environments; *cf*. the analogies 1–5). Getting permissions for such arrangements is usually difficult (especially at early validation stages). Moreover, in order to satisfy *reproducibility* conditions, each experiment needs a tedious and time-consuming individual setup (*cf*. Assumption 2 and Ex. 2.3). Therefore, as an alternative (as a preliminary test phase) we propose:

1. A *Turing test*-like approach, in which the framework is used to create avatars (“digital twins”) residing in realistic virtual environments, where their simulated behavior is observed and validated by therapists or by early-detection computer tools; *cf*. (Dillion et al., 2023) and (Perochon et al., 2023), respectively.

Remark 4.1. Validation methods which are useful to assess higher-functioning individuals and which (implicitly) assume an individual is able to perceive abstracts and operate on them, *e.g.*, by examining their propensity to ignore *opportunity costs* (see *e.g.*, Da Silva et al., 2025), cannot be used here.

### 4.2 Final remarks

We conclude the work with a claim that reducing uncertainty is not specific to autism.

Conjecture 4.1. The phenomenon of *insistence on sameness* in autism is a special case of the generic behavior pattern described in Friston (2010) where “*Adaptive agents must occupy a limited repertoire of states and therefore minimize the long-term average of surprise associated with sensory exchanges with the world* [and that] *action under the free-energy principle reduces to suppressing sensory prediction errors that depend on predicted (expected or desired) movement trajectories*.”

Therefore, a distinctive character of *insistence on sameness* should rather be attributed to cognitive limitations of a person with autism. Thus, if correct, the claim corroborates the following definition of the disorder; *cf*. (Mottron and Bzdok, 2020), Assumptions 2–3 and Remark 2.1:

Definition 4.1. Autism is an impairment in which cognitive functions are restricted to discrimination, memorization and prediction of tangible properties of the environment.

## Data Availability

The original contributions presented in the study are included in the article/supplementary material, further inquiries can be directed to the corresponding author.
